# Approaches to increase the validity of gene family identification using manual homology search tools

**DOI:** 10.1007/s10709-023-00196-8

**Published:** 2023-10-10

**Authors:** Benjamin J. Nestor, Philipp E. Bayer, Cassandria G. Tay Fernandez, David Edwards, Patrick M. Finnegan

**Affiliations:** 1 School of Biological Sciences, University of Western Australia, Perth, WA 6009 Australia; 2 Centre for Applied Bioinformatics, University of Western Australia, Perth, WA 6009 Australia

**Keywords:** Gene family identification, Homology, Genome analysis, Sequence similarity, Sequence evolution

## Abstract

Identifying homologs is an important process in the analysis of genetic patterns underlying traits and evolutionary relationships among species. Analysis of gene families is often used to form and support hypotheses on genetic patterns such as gene presence, absence, or functional divergence which underlie traits examined in functional studies. These analyses often require precise identification of all members in a targeted gene family. Manual pipelines where homology search and orthology assignment tools are used separately are the most common approach for identifying small gene families where accurate identification of all members is important. The ability to curate sequences between steps in manual pipelines allows for simple and precise identification of all possible gene family members. However, the validity of such manual pipeline analyses is often decreased by inappropriate approaches to homology searches including too relaxed or stringent statistical thresholds, inappropriate query sequences, homology classification based on sequence similarity alone, and low-quality proteome or genome sequences. In this article, we propose several approaches to mitigate these issues and allow for precise identification of gene family members and support for hypotheses linking genetic patterns to functional traits.

## Background to gene family identification

Recent increases in the number and quality of sequenced genomes has allowed in-depth comparison of genes between species and individuals through both single reference genomes and multiple species pangenomes (Bayer et al. 2020; Fernandez et al. 2022a). Genes shared between species or closely-related genes in the same species are known as homologs. Homologs known as orthologs originate from a common ancestral gene due to speciation events, while homologs known as paralogs arise from gene duplication in the same species (Fitch 1970; Setubal and Stadler 2018; Glover et al. 2019; Nevers et al. 2020). Identification of the homologs in gene families may take a whole genome approach where many different gene families and homologous members are identified, or a targeted approach where homologs of a specific gene family are identified with high accuracy. In both cases, genes translated from open reading frames to protein sequences are assigned as candidate homologs based on various measures of identity to protein sequences that are already characterised in those families from the same or different species. Sequences are usually classified as candidate homologs if the similarity between translated protein sequences is greater than that expected by chance (Pearson 2013; de Boissier and Habermann 2020). Candidate homologs can also be identified by the presence of conserved sequence regions such as motifs or functionally characterised domains in cases where exon shuffling, sequence rearrangements and modification, or gene fusion events cause low overall sequence identity between homologous sequences (Buljan and Bateman 2009; Forslund et al. 2011; Wu et al. 2012; Gabaldón and Koonin 2013). Homolog identification forms the basis for many downstream analyses in genome exploration such as analysis of trait and gene correlation, gene expression, gene functional mutation, gene ontology-based functional enrichment, phylogenetics, protein structure modelling, and comparative genomics. This diversity of applications reinforces the need for accurate homology searches whether by whole genome or targeted approaches.

An important use of identifying homologs, specifically orthologs, is that the function of an uncharacterised protein sequence can be hypothesised by its relationship to an ortholog that is already functionally characterised. Relationships of orthologs are most usefully derived from model species with many functionally characterised genes such as *Arabidopsis thaliana,* rice (*Oryza sativa*), filamentous fungi (*i.e. Aspergillus nidulans*), mouse (*Mus musculus*), fruit fly (*Drosophila melanogaster*), or *Escherichia coli*. However, several studies have challenged the assumption that orthologs always have similar functions, particularly for orthologs in different species with high evolutionary distance. Protein sequence similarity alone does not indicate that sequences will share the same function and expression patterns of this function because high similarity can result from conserved sequence domains or other low-complexity regions (Pearson 2013; Sinha et al. 2018; Stamboulian et al. 2020). Hence, while functional characterisation of genes based on sequence similarity provides a basis for hypothesising gene functions, confirmation of these functions is needed through gene expression or other functional analyses.

The identification of gene families in genomes is often used to form or support hypotheses of functional studies based on the genes present and evolutionary relationships of species. These studies are particularly important where large scale analysis of complex traits is needed or where functional studies such as mutant studies would fail because of the inability to examine mutations of vital or functionally-redundant genes (Favre et al. 2014). An example of the use of homolog identification is in the linking of genetic patterns such as presence or absence of homologs, gene family size, protein structure, or conservation of sequence motifs, functional sites, and residues to functional traits among different species (Khan et al. 2016; Leelananda and Lindert 2016; Glover et al. 2019). Ideally, genetic patterns at specific genomic regions are compared between genomes of a species with the functional trait and a closely-related species lacking the functional trait to minimise genetic differences arising from evolutionary distance. Differences in genetic patterns between these species can then be hypothesised as a potential mechanism that underlies the trait (Huynen et al. 1998; Jim et al. 2004; Nevers et al. 2020). Functional studies such as this have been used to identify genes involved in symbioses with arbuscular mycorrhizae (Delaux et al. 2014; Favre et al. 2014) and symbioses with nitrogen-fixing bacteria (Mergaert et al. 2020; Radhakrishnan et al. 2020). Similar studies have also been performed in prokaryotic microorganisms to predict genes associated with temperature-dependent virulence (Bocsanczy et al. 2017) and gene patterns linked with flagella, pili and thermophily (Jim et al. 2004). However, the validity of analyses that involve homolog identification greatly depend on the accuracy of this identification. A high accuracy of homolog identification is particularly important where a gene is hypothesised to be absent from a genome because gene family members may easily be missed in identification steps.

## Automated and manual pipelines for gene family identification

Many automated and manual pipelines for homology searches have been tested and benchmarked in services such as the Quest for Orthologs (Nevers et al. 2022). Well known examples of automated pipelines include Ortho-Markov Cluster Algorithm (OrthoMCL) (Li et al. 2003), Protein Annotation Through Evolutionary Relationship (PANTHER) (Thomas et al. 2003), and OrthoFinder (Emms and Kelly 2019) which have been reviewed extensively (see Glover et al. 2019; de Boissier and Habermann 2020; Nevers et al. 2020). Automated pipelines are generally used to rapidly compare large datasets for whole genome approaches such as genome annotation and the results from using different tools can easily be compared. However, this ability to compare large datasets comes at the cost of requiring a large amount of bioinformatic user-skill, computational power, and in-depth knowledge of tool usage to achieve precise identification of all gene family members without inclusion of members in other gene families (Steinegger et al. 2019; de Boissier and Habermann 2020; Nevers et al. 2020). Furthermore, automated pipelines trade the ability to manually curate homologous sequences between steps of the pipeline in favour of analysis speed and ease of use (Habermann 2016). Automated pipelines for homology search are useful for whole genome analyses, but often fall short for precise identification of gene families where the presence or absence of members must be confirmed with high confidence.

In manual pipelines, major steps are performed separately with simple homology-search tools. These steps often include a homology search tool to identify candidate homologs, usually predicted protein sequences, followed by sequence alignment and phylogenetic analysis. In brief, protein sequences with high similarity to query sequences in the targeted gene family are identified using homology search tools such as the Basic Local Alignment Search Tool (BLAST) (Altschul et al. 1990; Camacho et al. 2009) or Hidden Markov Modeler (HMMER) (Eddy 2011). Matching sequences that pass a fixed threshold value of significance are extracted (Nevers et al. 2020). These protein sequences are aligned using a multiple sequence aligner such as MUltiple Sequence Comparison by Log-Expectation (MUSCLE) (Edgar 2004) or Multiple Alignment using Fast Fourier Transform (MAFFT) (Katoh and Standley 2013). Matching protein sequences are compared to characterised members of the targeted gene family in a phylogenetic tree constructed with tools such as Randomized Axelerated Maximum Likelihood (RAxML) (Stamatakis 2014) or MrBayes (Ronquist et al. 2012). If the matching sequences appear to be part of the targeted gene family based on alignment and phylogenetic grouping, they are then reported as candidate homologs.

Manual pipelines have the advantage of allowing output sequences to be curated by the user at each step, greatly reducing the errors in the gene family members that are reported and in associated downstream analyses. Manual pipelines that incorporate conserved domain search tools as well as sequence similarity search tools are particularly useful for identifying remote homologs. Remote homologs are sequences that have low protein sequence similarity, making them difficult to detect by automated pipelines that generally use sequence similarity searches to identify homologs (Rost 1999; Habermann 2016; de Boissier and Habermann 2020). One downside to manual pipelines in comparison to automated pipelines is that the separation of steps comes at the cost of increased analysis time and reduced ability to compare results between different tools. However, the advantages of manual pipelines in precise gene family member identification leads them to be widely used for analyses of small targeted gene families.

Manual pipelines are commonly used to identify members of gene families in publications documenting the assembly of newly assembled genomes (Dong et al. 2021; Feng et al. 2021; Huang et al. 2021a; Li et al. 2021; Rai et al. 2021; Wang et al. 2021a; Apablaza et al. 2022). When implementing a manual pipeline to identify a gene family, it is important to consider several issues that affect the confidence in the homologs that are reported. Common issues in manual pipelines include too relaxed or stringent statistical thresholds, inappropriate query sequences, lack of multiple homology search tools, and low-quality proteome sequences (Pearson 2013; Sinha and Lynn 2014; Habermann 2016; Nevers et al. 2020). These issues can lead to exclusion of authentic homologs (false negatives) or the inclusion of inauthentic homologs from different gene families (false positives). False negatives and false positives can lead to poor support for hypotheses being tested and the propagation of errors in analyses of sequences, genetic mechanisms involved in functional traits, and evolutionary relationships among species. The perceived validity and reproducibility of the analyses is also weakened if the homology search process is not thoroughly documented in the research methods. Nevertheless, manual pipelines combined with strong justification of their use and methods are simple and precise tools for identifying gene family members and forming hypotheses. Many different variations of manual pipelines have been developed over the years. Here, we highlight the issues of manual pipelines in accurately identifying candidate homologs and review current approaches in the literature used to overcome these issues and to accurately identify gene family members with high confidence.

## Sequence similarity searches using Basic Local Alignment Search Tool (BLAST)

Studies implementing manual pipelines for gene family identification often use the extensively-used homology search tool BLAST, which depends on sequence similarity (Li et al. 2021; Patiranage et al. 2021; Pei et al. 2021; Rai et al. 2021; Wang et al. 2021a; Xu et al. 2021; Zhang et al. 2021; Zhao et al. 2021; Zhong et al. 2021). BLAST uses small local alignments between a query and target sequence to find regions of sequence identity or similarity known as hits scored by pre-defined matrices (Altschul et al. 1990; Pearson 2013). Several statistics are provided in the output of BLAST to help determine if a hit is part of an authentic homolog, including the E-value, alignment length, alignment coverage, and percent identity between the hit and query sequence. The E-value is a statistical score of significance that can be explained as the number of high scoring hits that would be found simply due to random combinations of nucleotides or amino acids in the target sequence that match the query sequence (Wheeler and Bhagwat 2007; Pearson 2013). Hence, a low E-value indicates that a hit is statistically significant and provides evidence that an authentic homolog was identified in the BLAST search. E-value thresholds for statistical significance are usually set in the range of 1e^−2^ to 1e^−20^ (Wheeler and Bhagwat 2007; Pearson 2013; Setubal and Stadler 2018; Miao et al. 2021; Rai et al. 2021). However, no E-value threshold is applicable for all analyses because E-values are dependent on the size of the target database and the size of query and target sequences. Thresholds of other BLAST output statistics such as alignment coverage above 50–80% and percent identity above 50% can also be used to provide further evidence that hits are authentic homologs (Li et al. 2021; Pei et al. 2021). Where the lengths of typical homologs in the targeted gene family are known, BLAST hits can be filtered by length of alignment or length of the extracted open reading frame (ORF) to avoid shortened pseudogenes or sequences that match only part of the query sequence being included (Niu et al. 2021; Rai et al. 2021; Wang et al. 2021b). By carefully selecting thresholds for E-values, coverage, percent identity, and sequence length to filter BLAST hits, greater confidence can be gained that the selected BLAST hits are authentic homologs.

**Fig. 1:**
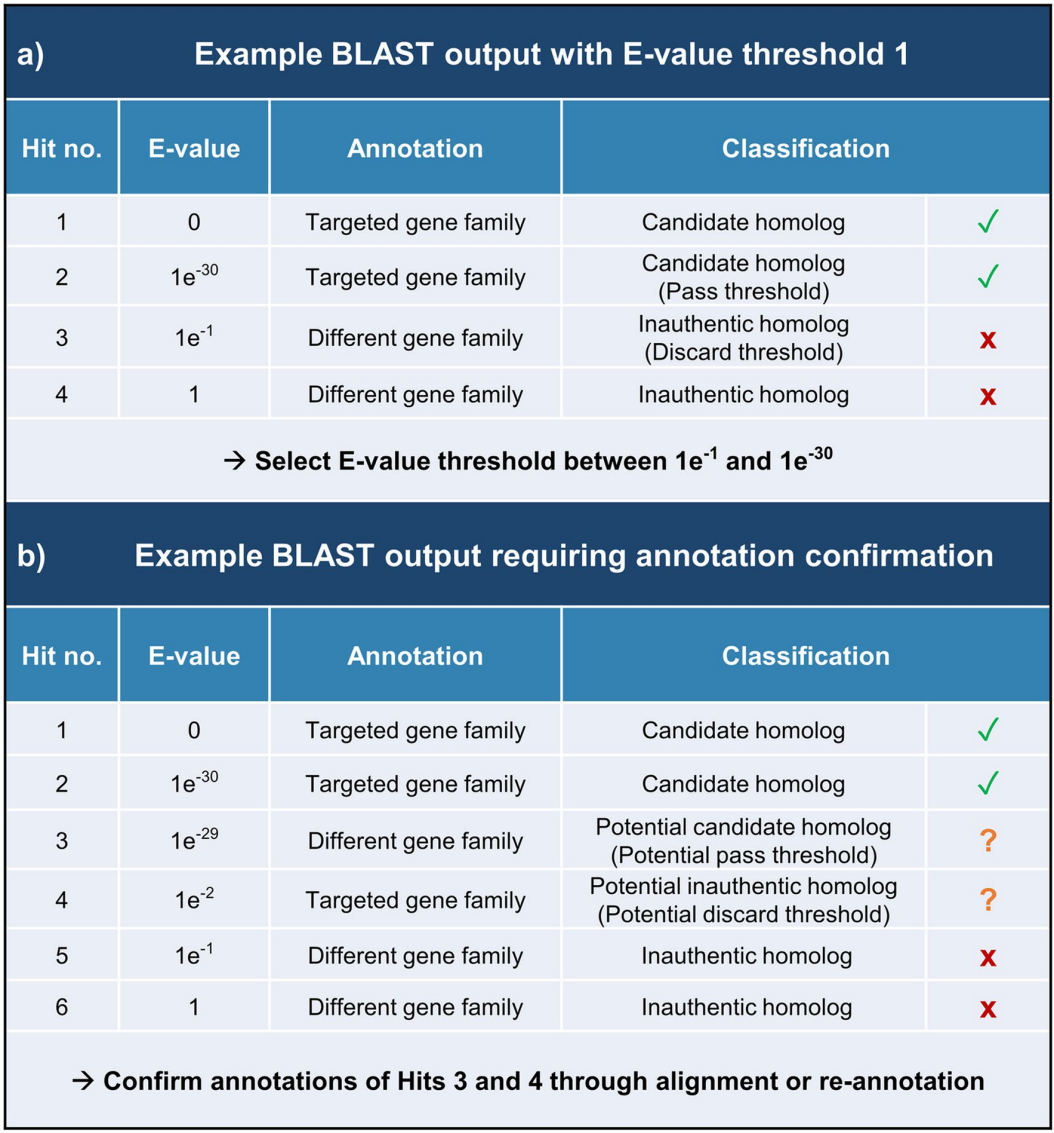
Process for reporting BLAST hits as candidate homologs with high confidence using a pre-selected E-value based on pass and discard thresholds. **a** An E-value threshold between 1e^−1^ and 1e^−30^ is selected (1e^−20^ for example) based on the pass and discard threshold E-values of BLAST hits. **b** Confirmation of sequence annotations is needed before selection of an E-value threshold. In this case, the potential pass threshold sequence is annotated in a different gene family, while the potential discard threshold sequence is annotated in the targeted gene family

The foremost issue with BLAST homology searches is that too stringent E-value thresholds in BLAST searches can lead to false negatives, where authentic homologs are missed, while too relaxed thresholds can lead to false positives, where inauthentic homologs are retained (Pearson 2013; Fujimoto et al. 2016; Habermann 2016; de Boissier and Habermann 2020). Low E-value thresholds are commonly used as the only threshold for determining if BLAST hits are authentic homologs, resulting in a high possibility for false negatives and a low confidence in any genes reported missing from the targeted genome. However, a higher confidence in BLAST searches can be achieved through a step-by-step process to pre-determine an appropriate E-value (Fig. 1). Firstly, a relatively high E-value between 1 and 10 is used to retrieve all potential hits of matching protein sequences with a high likelihood for false positives. The annotations of these protein sequences are then examined to identify a pass and a discard E-value threshold. The pass threshold is the highest E-value of a protein sequence annotated to be in the targeted gene family while the discard threshold is the lowest E-value of a protein sequence annotated as not in the targeted gene family. If the pass threshold E-value is relatively high compared to the E-values of other genes in the targeted gene family, or the discard threshold E-value is relatively low compared to E-values of other discarded genes, then the annotations of these sequences should be re-confirmed by BLAST searches to the National Center for Biotechnology Information (NCBI) non-redundant (NR) database (Sayers et al. 2022) or alignment with other protein sequences in the targeted gene family. A final E-value threshold between the pass and discard thresholds and if applicable also between the typical E-value range of 1e^−2^ to 1e^−20^ can then be chosen to filter the BLAST output and ensure that there are no false negatives or false positive homologs. In literature reports, the chosen E-value and the pass and discard threshold E-values should be reported as well as the annotations of the pass and discard threshold sequences to increase reader confidence in reported candidate homologs.

We have provided example data for choosing an E-value threshold during a BLAST homology search (Table 1). Here, BLAST searches were performed against the NCBI RefSeq predicted protein sequence database for *Nelumbo nucifera* (lotus) to identify members of the *PHOSPHATE TRANSPORTER 1* (*PHT1*) and *PHOSPHATE1* (*PHO1*) gene families. These gene families are important for phosphate transport in plants and are frequently the subject of homology searches involving plant genomes. In this example, all sequences annotated as PHT1 that were retrieved from the *N. nucifera* database by BLAST using 20 PHT1 protein query sequences from the model plant species *Arabidopsis thaliana* and *Oryza sativa* (rice) had an E-value of 0. The next best match, which belonged to another gene family, had an E-value of 6.05e^−14^. Hence, an E-value threshold below 6.05e^−14^, such as 1e^−20^, would be appropriate to retrieve homologs in this homology search. However, the situation was different for the identification of PHO1 protein sequences. Almost all sequences annotated as PHO1 that were retrieved from the *N. nucifera* database by BLAST using 14 PHO1 protein query sequences from *A. thaliana* and *O. sativa* had an E-value of 0, indicating that they are likely true homologs. In contrast, the next best match was to a non-PHO1 family member which had an E-value of 9.74e^−34^. Therefore, using the E-value threshold of 1e^−20^ as in the PHT1 search would lead to false positives and be inappropriate for this homology search. In addition, a short protein sequence 90 amino acids in length with a PHO1 annotation was recovered that had an E-value of 9.34e^−10^. This sequence would have been discarded if an E-value threshold above 9.74e^−34^ was used. In order to demonstrate that no *N. nucifera* PHO1 protein sequence homologs have been missed in the analysis, this short protein sequence annotated as PHO1 must be examined further to determine whether it can be filtered out due to its low sequence length or low alignment coverage, or whether the sequence is a potentially important PHO1 ortholog to be considered further in the study at hand. Once the outlier sequence has been kept or discarded, the E-value threshold can be set below 9.74e^−34^, such as 1e^−40^, to retrieve homologs for further analysis. An E-value threshold of 1e^−40^ is seemingly quite low, but is still appropriate for the PHO1 analysis since sequence hits with lower E-values have been checked to increase the confidence that there are no false negatives or positives.

**Table 1:**
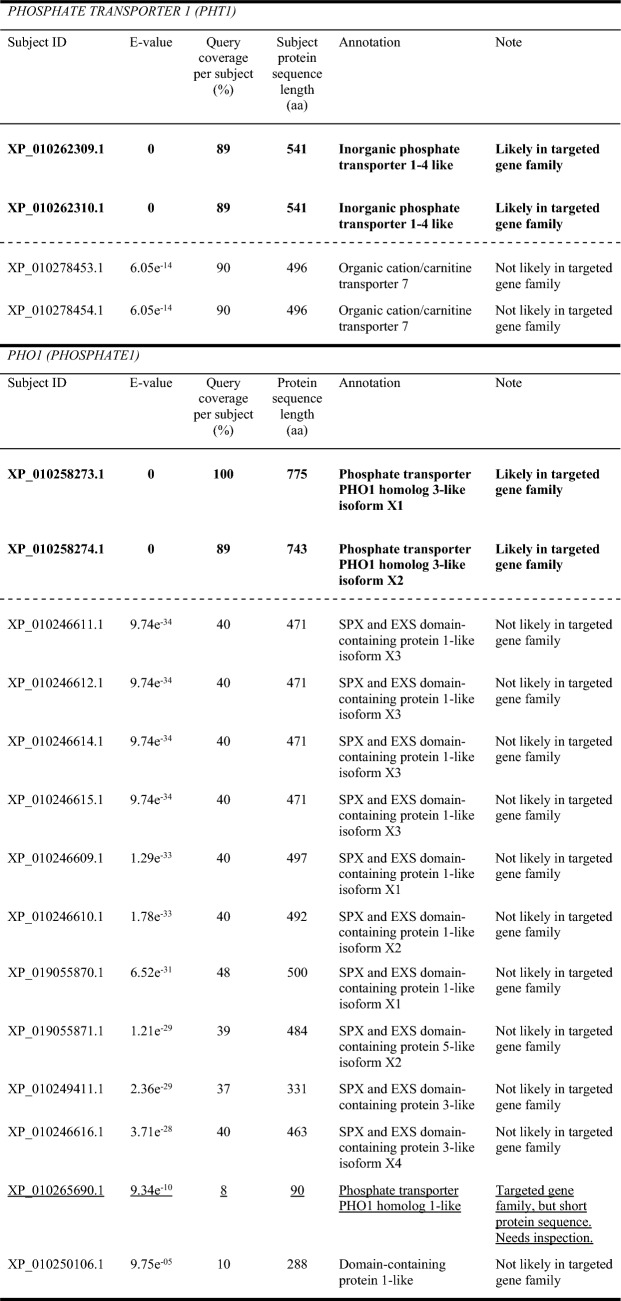
Example data for choosing an E-value threshold in the NCBI RefSeq predicted protein sequence database of *Nelumbo nucifera* (GenBank accession: GCF_000365185.1). Members of the *PHOSPHATE TRANSPORTER 1* (*PHT1*) and *PHOSPHATE1* (*PHO1*) gene families were identified using BLAST (v. 12.2.0) with query protein sequences from *Arabidopsis thaliana* and *Oryza sativa*

Query sequences from *Arabidopsis thaliana* and *Oryza sativa* were retrieved from the Araport11 protein sequence database through The Arabidopsis Information Resource (Berardini et al. 2015) and the Swiss-Prot database, UniProtKB (The Uniprot Consortium 2015). PHT1: AT5G43350, AT5G43370, AT5G43360, AT2G38940, AT2G32830, AT5G43340, AT3G54700, AT1G20860, AT1G76430, Q7XDZ7, Q01MW8, Q7X7V2, Q8H6H0, Q8H6G9, Q8H6G8, Q8H6G7, Q69T94, Q8H6H4, Q8GSD9, Q7XDZ7. PHO1: Q8S403, Q93ZF5, Q6R8G8, Q6R8G7, Q6R8G6, Q6R8G5, Q6R8G4, Q6R8G3, Q6R8G2, Q9LJW0, Q6R8G0, Q657S5, Q6K991, Q651J5

Sequences likely to be authentic homologs are highlighted in bold, and potential homologs that require further examination are underlined. A dotted line is placed between the pass and discard E-values for both homology searches and indicates the range where an appropriate E-value threshold can be set. For simplicity, the results have been trimmed to show only sequence hits with E-values surrounding the pass and discard thresholds and potential homologs requiring further investigation

In cases where homologs are short or have little similarity to a query sequence, known as remote homologs (Habermann 2016; Yang et al. 2021), a more diverse group of query sequences can increase the accuracy of BLAST searches. Remote homologs often still share sequence similarity due to conserved protein structure or conserved domains of the targeted gene family, meaning they will likely be missed by strict E-value cut-offs in BLAST, which matches protein sequences based only on high sequence similarity (Pearson 2013; Sinha and Lynn 2014; Habermann 2016). These remote homologs are more likely to be captured if using a diverse range of query sequences as this will represent the variation present in the targeted gene family. In recent studies on gene family identification in plants (Liu et al. 2021; Rai et al. 2021), sequences from up to 15 species have been used as BLAST query sequences. If using a single or very small set of homologs, large collections from NCBI NR, the Universal Protein Resource (UniProt; https://www.uniprot.org/) (The Uniprot Consortium 2015), or Ensembl (Cunningham et al. 2021) could also be used (Liu et al. 2021). Clustered protein sequence databases such as UniRef (Suzek et al. 2015) provide a smaller sequence subset for selecting queries or confirming identified homologs that still maintains a high level of sequence diversity for remote homolog detection. UniRef contains clustered protein sequences from UniProt based on sequence identities ranging from 50% (UniRef50) to 100% (UniRef100). These clusters can be used to select a diverse range of protein sequences as query sequences, or as a database to investigate the annotations of sequences that cluster with identified sequences. The use of more diverse query sequences selected from a wide range of species or clustered databases may increase computational requirements for homology searches, but will greatly increase the confidence that can be given to homology searches based on sequence similarity.

In many cases, a targeted gene family will be too large to use queries from multiple species. Here, queries specific to the targeted species can be generated using Position-Specific Iterative BLAST (PSI-BLAST) (Altschul et al. 1997). In PSI-BLAST searches, an initial BLAST search retrieves a list of high-scoring hits to sequences based on an E-value threshold, and these sequences are then used to produce an alignment and a position-specific scoring matrix (PSSM) (Altschul et al. 1997; Sinha et al. 2018). Residue scores in the PSSM are used for iterative similarity searches to the target sequences with the scores modified after each iteration based on alignment hits that pass a threshold E-value. PSI-BLAST can be useful for identifying divergent sequences because the generated PSSM is designed specifically for the target proteome and gene family (Altschul et al. 1997; Andolfo et al. 2021). The PSI-BLAST approach can be used as an alternative to a large set of query species for greater confidence that all members of the targeted gene family have been identified.

## Profile domain searches using Hidden Markov Modeler (HMMER)

A second commonly used method for homology searches is HMMER (Andolfo et al. 2021; Dong et al. 2021; Feng et al. 2021; Guérin et al. 2021; Huang et al. 2021a; Qin et al. 2021; Wu et al. 2021; Apablaza et al. 2022). HMMER is used to search for homologs based on conserved sequence domains rather than sequence similarity, which allows identification of remote homologs with low overall sequence similarity (Rost 1999; Habermann 2016; de Boissier and Habermann 2020). Sequence domains in a gene family that are conserved across many species often have functional importance and so these domains are expected to be detectable in the majority of homologs in the gene family (Richardson 1981; Ghouila et al. 2014; Lees et al. 2016). HMMER identifies conserved domains based on probabilistic models of sequences known as profile Hidden Markov Models (profile HMMs) (Eddy 1998). Like BLAST, HMMER outputs an E-value statistic to aid in determining if target sequences are authentic homologs of the targeted gene family. The E-value in this case refers to the expected number of false positive sequences being included, with the E-value increasing as dataset size increases. An appropriate range for the threshold of this E-value can be determined through the method used for BLAST (Fig. 1). HMMER is often used in combination or as an alternative to BLAST and both programs can be highly adept at detecting authentic homologs if used with appropriate thresholds.

HMMER can be combined with BLAST by using the candidate homologs of one program as the target sequences for the next program (Liu et al. 2021; Yan et al. 2021), or by using both tools simultaneously and then filtering for common sequences (Pareek et al. 2021; Wang et al. 2021a). Similar to BLAST searches, it is important to generate profiles from several phylogenetically-related species or use family-specific profile HMMs from the target species or the Pfam database (Mistry et al. 2021) through InterPro (Paysan-Lafosse et al. 2022). Using suitable profiles will reduce false negatives where homologs are evolutionarily distant or have divergent domain structures (Ghouila et al. 2014) and also reduce false positives where domains in the profile HMM are not specific to the target gene family (Sinha et al. 2018). Extensive profile HMMs for conserved domains of diverse groups of sequences can be downloaded from Pfam or new profiles can be generated from a user's own sequence sets to identify matching sequences with the same sequence domains. Homologs can be further confirmed by searching other conserved domain and signature databases such as Simple Modular Architecture Research Tool (SMART) (Schultz et al. 1998), the Conserved Domain Database (CDD) using CD-Search (Marchler-Bauer and Bryant 2004), or PROSITE (Sigrist et al. 2012). Greater control of conserved domain detection and use of user-made motifs can be achieved following a similar method to Andolfo et al. (2021) where Multiple EM for Motif Elicitation (MEME) was used to extract motifs from Pfam domains and these were then searched in target sequences using Motif Alignment and Search Tool (MAST) (Bailey et al. 2015). Several protein family databases including PANTHER, CDD, Pfam, SMART, and PROSITE, among other useful protein motif, domain, signature and site databases, can also be searched simultaneously using InterPro through InterProScan (Paysan-Lafosse et al. 2022) to provide a comprehensive analysis of potential orthology for identified protein sequences. In summary, protein family database searches available through tools such as HMMER or InterProScan are powerful tools alone or in combination with BLAST for accurate identification of homologs in gene families.

## Confirming genes in the genome sequence

A common source of false negatives and false positives in homology searches is from low-quality predicted proteome databases derived from genome sequences. Low-quality predicted proteomes often contain a high proportion of fragmented, chimeric, or contaminant protein sequences that arise due to errors in genome sequencing or in the assembly and gene prediction stages (Li et al. 2006; El-Metwally et al. 2013; Richards 2018). False negatives will occur if fragments or chimeric sequences belonging to authentic homologs contain enough errors that they are undetectable by query sequences or profile HMMs (Nevers et al. 2020). On the other hand, false positives will occur if inauthentic homologs are represented as fragmented or chimeric sequences, of which a large proportion of the sequence is a low complexity or conserved domain region and has a strong match in the homology search. To alleviate both these issues, it is important to use high-quality genome and predicted proteome databases such as reference proteomes that are available through UniProt. Recent advances in genome sequencing such as long read sequencing and improvements in genome assembly and annotation tools have made high-quality genome and predicted proteome databases readily available for many species (Angel et al. 2018; Rice and Green 2019; Fernandez et al. 2022a). However, high-quality proteomes are still lacking for many non-model species, meaning homolog identification in these species must be accompanied by high-quality genome assembly and proteome prediction. Using these sources of high-quality proteomes will greatly reduce the chance of false negatives and false positives in reported gene family members.

In some cases, high-quality proteome databases are not available and additional verification is needed to demonstrate the presence or absence of gene family members in the genome. Even when an in-depth homology search is performed, authentic homologs may still be missed if the proteome being searched has a relatively low completeness score due to poor-quality gene prediction (Dohmen et al. 2016). Although the quality of sequencing and genome assembly methods are rapidly improving and associated costs are decreasing, most genome assemblies will still likely contain misassemblies due to base changes or larger insertions/deletions (indels), which often prevent annotation tools from correctly predicting genes (Watson and Warr 2019; Huang et al. 2021b; Fernandez et al. 2022b). However, these missed genes can be detected by examining the genome sequence of these species. Regions of the genome sequence that have sequence identity with the targeted gene family but lack a predicted gene can be extracted and ORFs predicted using gene prediction tools. The BLAST tblastn tool can be used to search all frame translations of the genome sequence for regions of sequence identity using protein query sequences. This method was used in Fernandez-Pozo et al. (2021) and in Marsh et al. (2023) to extract gene hits within over 1 kbp flanking regions and predict ORFs using Augustus (Stanke et al. 2006). Similar methods to predict ORFs were followed in Chen et al. (2021) using the BLAST-Like Alignment Tool (BLAT) (Ward and Moreno-Hagelsieb 2014) to detect potential homologs, and in Ji et al. (2021) using GeneWise (Birney et al. 2004). Other tools that can be used to predict genes in extracted nucleotide sequences include SNAP (Korf 2004) and Fgenesh (Salamov and Solovyev 2000). Alternatively, extracted regions with potential genes can be examined by alignment with the coding DNA sequence (CDS) of genes from the targeted gene family. Comparing tools for different gene predictions and genome sequence homology searches is difficult, because the results will largely differ depending on the target species and gene family, but implementing any form of the genome search and ORF prediction or alignment approach will lead to a higher confidence that all authentic homologs of a gene family have been identified.

## Final confirmation of candidate homologs

**Fig. 2:**
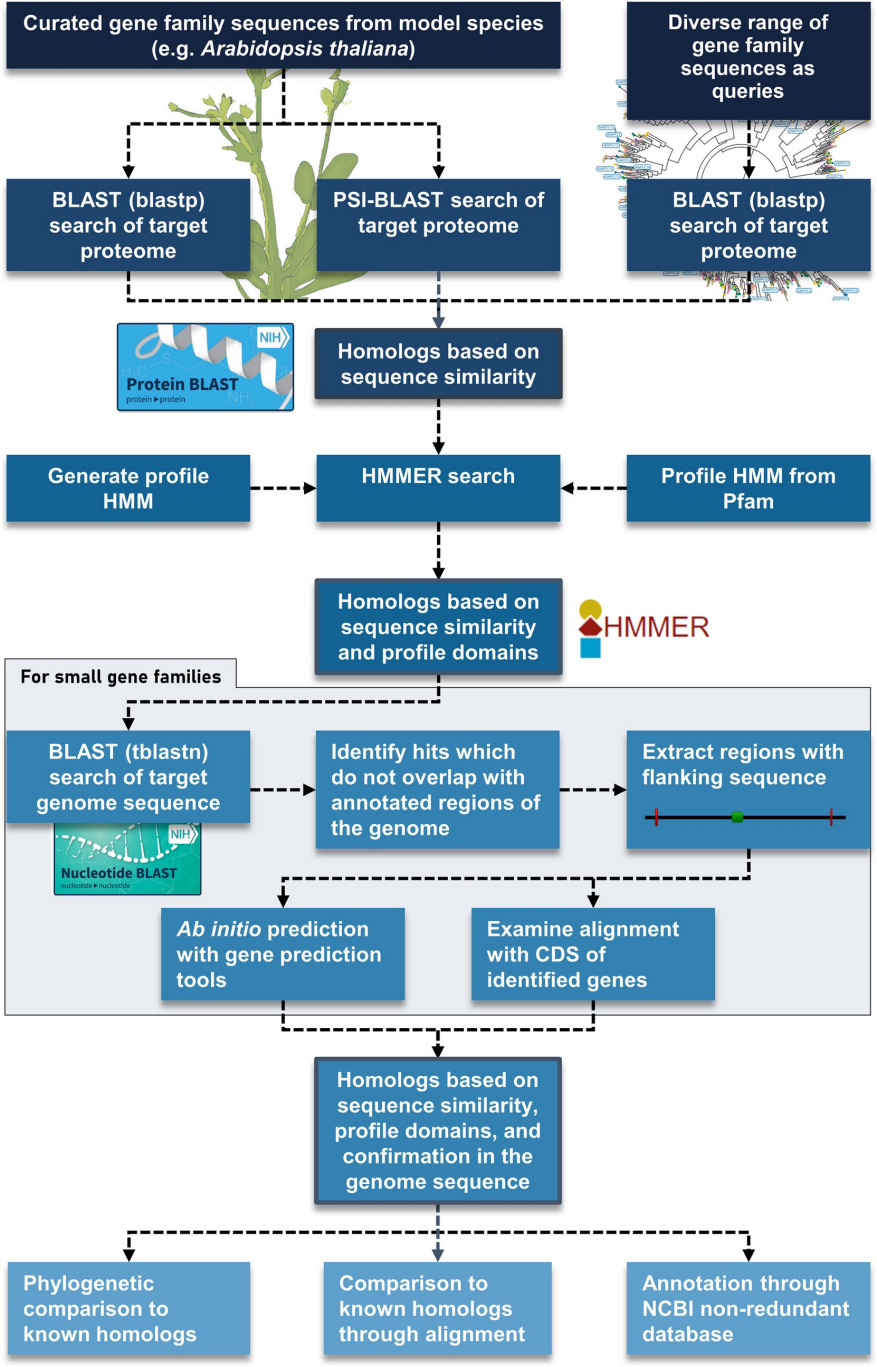
Suggested approach for homolog identification based on sequence identity and profile domains. Input sequences are either from model species or a diverse range of protein sequences using a phylogenetically diverse species range or clustered protein sequence database. These input sequences are compared with the target predicted proteome using Basic Local Alignment Search Tool (BLAST) protein (blastp) or Position-Specific Iterative BLAST (PSI-BLAST). Hidden Markov Modeler (HMMER) is then applied with a user-generated profile or downloaded Pfam profile to identify candidate homologs based on both sequence identity and profile domains. If the target gene family is relatively small, the genome sequence can be checked to ensure that no unannotated genes have been missed. In the genome sequence check, a translated BLAST nucleotide (tblastn) search of the genome sequence will identify potential unannotated genes that can be extracted along with flanking sequences. The potential genes can be validated by using gene prediction tools or alignment with the coding DNA sequence (CDS) of previously identified genes in the target gene family. After all candidate homologs have been identified, the protein translations are further confirmed by phylogenetic comparison and alignment to other known proteins in the same gene family. Confirmation can also be performed using a BLAST search of the National Center for Biotechnology Information (NCBI) non-redundant (NR) database and examination of the top hit annotations

Once candidate homologs are extracted in a homology search, it is important to further confirm them to ensure that they are part of the targeted gene family. The presence of false positives may be less detrimental than false negatives in this case because the absence of false negatives cannot be verified while false positives can often be detected and removed by several methods. Methods to detect false positives include aligning sequences and then removing those sequences that are inconsistent with known homologs (Cao et al. 2021; Niu et al. 2021; Zhang et al. 2021) or building phylogenetic trees and removing sequences that occur as single-member deeply-rooted clades or highly divergent branches lacking orthologs of the targeted gene family (Li et al. 2006; Thanki et al. 2018). A BLAST search of candidate homologs against a database such as NCBI NR or UniProt is also a useful method to confirm if the sequence is part of the target gene family based on the annotations of top hits (Fernandez-Pozo et al. 2021). For validating a large number of genes, CD-HIT (Fu et al. 2012) can be used to select candidate homologs that cluster with known proteins (Rai et al. 2021). A summarised pipeline of the approaches and options for mitigating the manual pipeline issues we have discussed in this article is provided in Fig. 2. Although the approaches will likely extend the time and complexity of homolog searches using manual pipelines, their use will greatly increase the validity and thoroughness of gene family member identification allowing greater confidence in gene family analyses and reporting of non-functional or absent genes.

## Summary

Manual pipelines are widely used to identify gene family members in targeted gene family studies with the goal of linking gene patterns to functional traits, but several issues often hinder the validity of reported gene families. We suggest several approaches to mitigate issues with manual pipelines and minimise the number of false negatives and false positives in analyses. The foremost issue is that false negatives and false positives often result from the use of strict thresholds such as E-values, without these threshold values being validated for the specific analysis. An appropriate E-value can be selected from a pass and discard threshold based on the annotations of matching sequences. Furthermore, inappropriate query sequences are often used in homology search tools, which can result in false negatives by excluding authentic homologs or the inclusion of false positive genes. Among the options for query sequence selection is the use of divergent query sequences from a wide range of phylogenetically diverse species or clustered protein sequence databases. In addition, combining similarity and conserved domain search tools can increase the ability to identify and validate all members of a gene family. False negatives and false positives also result when using low-quality predicted proteomes that may not include some protein sequences or contain fragmented and chimeric protein sequences. In these cases, missing genes can be confirmed by alignment or gene prediction of regions containing potential genes in the genome sequence. We believe that the issues and approaches detailed in this article are important to consider for analyses requiring precise identification of all members of a targeted gene family. Implementation of these approaches in manual homology searches will greatly increase the confidence in gene family identification and the ability for accurate down-stream analyses on relating gene presence and absence to traits in model and non-model species.

## Data Availability

Data sharing not applicable to this article as no datasets were generated or analysed during the current study.
